# Supplementary material for: Analysis of PPARγ Signaling Activity in Psoriasis
Source: Int J Mol Sci. 2021 Aug 10;22(16):8603. doi: 10.3390/ijms22168603 (PMC8395241; doi:10.3390/ijms22168603)
Supplement: Supplementary file 1 [file ijms-22-08603-s001.zip › Supplemental materials_Analysis of PPARg signaling activity in psoriasis/Pathway models/Models images and html files/PPARG inhibits phenotypes assotiated with psoriasis/28579.html]

inflammation


# Disease inflammation

|  |  |
| --- | --- |
| URN | urn:agi-meshdis:Inflammation |
| Total Entities | 40 |
| Connectivity | 24440 |
| Name | inflammation |

---

|  |  |
| --- | --- |
| ChildConcepts | phlegmon |
|  | neuroinflammation |
|  | granulomatous inflammation |
|  | cardiovascular inflammation |
|  | dermatitis |
|  | abdominal cavity inflammation |
|  | application site inflammation |
|  | inflammatory disease |
|  | mastitis |
|  | musculoskeletal inflammation |
|  | chronic inflammation |
|  | cytokine release syndrome |
|  | cytokine storm |
|  | gastrointestinal inflammation |
|  | eye and surrounding structures inflammation |
|  | urogenital tract inflammation |
|  | hemic and lymphatic system inflammation |
|  | airway inflammation |
|  | inflammatory infiltrate |
|  | cellulitis |
|  | injection site inflammation |
|  | oral inflammation |
|  | serositis |
|  | postoperative inflammation |
|  | sensory organ inflammation |
|  | soft tissue inflammation |
|  | experimental inflammation |
|  | acute phase response |
|  | foreign body reaction |
|  | immune reconstitution inflammatory syndrome |
|  | mediastinitis |
|  | systemic inflammatory response syndrome |
|  | neurogenic inflammation |
|  | omphalitis |
|  | inflammatory stress |
|  | leukocyte infiltration |
|  | mucositis |
|  | implant inflammation |
|  | necroinflammation |
|  | inflammatory lesion |

---

|  |  |
| --- | --- |
| Pathway | Proteins with Altered Expression in Aging |
|  | Necrosis |
|  | Lipids Enhance Apoptotic Cell Engulfment and Reduce Inflammation |
|  | Keep out Signal: LTF Inhibits Neutrophil Recruitment and Inflammation |
|  | Leukotriene Effect on Airway Smooth Muscle Response |
|  | Mast-Cells De Novo Synthesized Mediators via IgE Independent Signaling |
|  | Macrophage Related Iron Uptake and Release |
|  | CD16/CD14 Proinflammatory Monocyte Activation |
|  | anti-Inflammatory Function of Macrophage M2 Lineage |
|  | Nociception Expression Targets Signaling |
|  | NPY1R -> CRH/POMC Production |
|  | NTSR1 Expression Targets |
|  | CRH -> Synthesis of Corticosteroids |
|  | CCR1 Expression Targets |
|  | ADRA1A -> IL6 Production |
|  | ADRB1 -> Prostaglandin Generation |
|  | ADRA1 -> Prostaglandin Generation |
|  | BDKRB1/2 -> Prostaglandin Generation |
|  | BDKRB1/2 -> Interleukins Production |
|  | CNR1/2 -> IL1B/2/4/6/10 Production |
|  | DRD2 Expression Targets |
|  | GALR1/2/3 -> POMC/NPY Production |
|  | HRH2/4 -> IL6/10 Production |
|  | CHRM1 -> IL2 Production |
|  | OPRK -> Pain Perception |
|  | PTGIR -> IL6 Production |
|  | PTGER2/3 -> Inflammation-Related Expression Targets |
|  | HTR1 -> IL6 Production |
|  | HTR7 -> IL6 Production |
|  | HTR5 -> TNF Production |
|  | TACR1 -> TNF/IL6/IL8 Production |
|  | IL6 Expression Targets -> Nociception |
|  | IL1B Expression Targets -> Nociception |
|  | Leptin -> CD25/IL6/IL10 Production |
|  | CHRNA7 -> IL8 Production |
|  | NMDA Receptors -> Ca2+/CREB Activation/PGE2 Synthesis |
|  | P2RY11/13/14 -> IL8/10 Production |
|  | Reperfusion Injury in Myocardial ischemia |
|  | Graves Ophthalmopathy |
|  | Fatty Acids in Hypertension and Atherosclerosis Development |
|  | Antiphospholipid Antibodies in Endothelial Cell |
|  | Antiphospholipid Antibodies and Endothelial Cell Interaction |
|  | SERPINA1 Associated Liver Damage |
|  | TRPM7/8 in Pancreatic Adenocarcinoma |
|  | Airway Epithelial Cell Dysfunction |
|  | Diabetes Induced Periodontitis |
|  | Drugs Targets in Autistic Disoreder (Hypothesis) |
|  | IL6 Promotes Inflammation in Muscular Dystrophies |
|  | Muscular Dystrophies |
|  | Local Estrogen Production in Endometriosis |
|  | Oxidative Stress, All-Trans-Retinal and Lipofuscin Toxicity in AMD |
|  | ER Stress in Macular Degeneration |
|  | Aminoglycosides and Cisplatin Induced Ototoxicity (Mouse Model) |
|  | Cochlear Inflammation (Noise-Induced) |
|  | Glucocorticoid Resistance |
|  | Cyclosporine Induced Nephrotoxicity |
|  | STAT3 and NFkB Activate Inflammation-Induced Tumorigenesis |
|  | IL23A and IL17A Provoke Cancer-Associated Inflammation |
|  | Asbestosis- and Silicosis-Associated Inflammation Provokes Cancer Development |
|  | Proteins with Altered Expression in Tumor-Promoting Inflammation |
|  | N2 Neutrophils in Tumor-Promoting Inflammation and Tumor Progression |
|  | NFKB Signaling Activation by Blocking of Tumor Suppressors |
|  | ROS in Triggering Vascular Inflammation |
|  | cypa |
|  | PPARG inhibits phenotypes assotiated with psoriasis |
|  | Neighbors of inflammation |
|  | Figure 1\_Levothyroxine clinical effects |
|  | Figure 1\_Levothyroxine clinical effects |
|  | 1\_Local estrogen production stimulates ectopic endometrium proliferation |
|  | 2\_Oxidative stress, all-trans-retinal, and lipofuscin toxicity in agerelated macular degeneration |
|  | 3\_Endoplasmic reticulum stress in age-related macular degeneration |
|  | 2\_High levels of hepcidin block iron absorption in the gut |
|  | 3\_Impairment of iron release from splenic macrophages |
|  | 1\_Inflammatory mediators increase the production of hepcidin (HAMP) |
|  | 5\_The rationale for the treatment of anemia of chronic disease with erythropoietin |
|  | 4\_Inflammatory mediators inhibit GATA signaling in erythropoiesis |
|  | 2\_SERPINA1-associated liver damage |

---

|  |  |
| --- | --- |
| MedScan ID | 9015255 |

---

|  |  |
| --- | --- |
| Alias | Inflammations |
|  | inflammatory syndromes |
|  | inflammatory conditions |
|  | inflammatory activity |
|  | inflammatory syndrome |
|  | Inflammation |
|  | inflammation reaction |
|  | acute inflammation |
|  | inflammatory process |
|  | inflammatory activities |
|  | Inflammatory Reactions |
|  | inflammatory condition |
|  | inflammatory processes |
|  | inflammatory processing |
|  | Inflammatory Reaction |
|  | inflammation reactions |

---

|  |  |
| --- | --- |
| MeSH ID | D007249 |
|  | M0011307 |

---

|  |  |
| --- | --- |
| MeSH Heading | Inflammation |

---
